# Apixaban in patients at risk of stroke undergoing atrial fibrillation ablation

**DOI:** 10.1093/eurheartj/ehy176

**Published:** 2018-03-20

**Authors:** Paulus Kirchhof, Karl Georg Haeusler, Benjamin Blank, Joseph De Bono, David Callans, Arif Elvan, Thomas Fetsch, Isabelle C Van Gelder, Philip Gentlesk, Massimo Grimaldi, Jim Hansen, Gerhard Hindricks, Hussein R Al-Khalidi, Tyler Massaro, Lluis Mont, Jens Cosedis Nielsen, Georg Nölker, Jonathan P Piccini, Tom De Potter, Daniel Scherr, Ulrich Schotten, Sakis Themistoclakis, Derick Todd, Johan Vijgen, Luigi Di Biase

**Affiliations:** 1 Institute of Cardiovascular Sciences, University of Birmingham, and SWBH and UHB NHS Trusts, IBR 136, Wolfson Drive, Birmingham, UK; 2 SWBH NHS Trust, Birmingham, UK; 3 University Hospitals Birmingham, Birmingham, UK; 4 Atrial Fibrillation NETwork Association (AFNET), Germany, Münster; 5 Center for Stroke Research Berlin & Department of Neurology, Charité – Universitätsmedizin Berlin, Germany; 6 Hospital of the University of Pennsylvania, PA, USA; 7 Isala Heart Center Zwolle, Zwolle, The Netherlands; 8 The Clinical Research Institute, Munich, Germany; 9 University of Groningen, University Medical Center Groningen, Groningen, The Netherlands; 10 Sentara Cardiovascular Research Institute, Norfolk, VA, USA; 11 Ospedale Generale Regionale F. Miulli, Acquaviva delle Fonti, Italy; 12 Gentofte Hospital, Hellerup, Denmark; 13 Abteilung für Rhythmologie, Leipzig Heart Center, Leipzig, Germany; 14 Department of Biostatistics & Bioinformatics, Duke University School of Medicine, USA; 15 Duke Clinical Research Institute (DCRI), Durham, NC, USA; 16 Hospital Clinic Barcelona, University of Barcelona, Barcelona, Spain; 17 Department of Cardiology, Aarhus University Hospital, Denmark; 18 Herz- und Diabeteszentrum NRW, Ruhr-Universität Bochum, Bad Oeynhausen, Germany; 19 Division of Cardiology Duke University Medical Center, Duke University, Durham, NC, USA; 20 Cardiovascular Center, OLV Aalst, Belgium; 21 Department of Cardiology, Medical University Graz, Austria; 22 Department of Physiology, University Maastricht, Maastricht, Netherlands; 23 Ospedale Dell'Angelo, Mestre, Italy; 24 Liverpool Heart and Chest Hospital, Liverpool, UK; 25 Jessa Ziekenhuis, Campus Virga Jesse, Hasselt, Belgium; 26 Albert Einstein College of Medicine, at Montefiore Hospital, New York, USA; 27 Texas Cardiac Arrhythmia Institute at St. David’s Medical Center, Austin, TX, USA

**Keywords:** Atrial fibrillation, Ablation, Anticoagulation, Bleeding, Stroke, Brain MRI

## Abstract

**Aims:**

It is recommended to perform atrial fibrillation ablation with continuous anticoagulation. Continuous apixaban has not been tested.

**Methods and results:**

We compared continuous apixaban (5 mg b.i.d.) to vitamin K antagonists (VKA, international normalized ratio 2–3) in atrial fibrillation patients at risk of stroke a prospective, open, multi-centre study with blinded outcome assessment. Primary outcome was a composite of death, stroke, or bleeding (Bleeding Academic Research Consortium 2–5). A high-resolution brain magnetic resonance imaging (MRI) sub-study quantified acute brain lesions. Cognitive function was assessed by Montreal Cognitive Assessment (MoCA) at baseline and at end of follow-up. Overall, 674 patients (median age 64 years, 33% female, 42% non-paroxysmal atrial fibrillation, 49 sites) were randomized; 633 received study drug and underwent ablation; 335 undertook MRI (25 sites, 323 analysable scans). The primary outcome was observed in 22/318 patients randomized to apixaban, and in 23/315 randomized to VKA {difference −0.38% [90% confidence interval (CI) −4.0%, 3.3%], non-inferiority *P *= 0.0002 at the pre-specified absolute margin of 0.075}, including 2 (0.3%) deaths, 2 (0.3%) strokes, and 24 (3.8%) ISTH major bleeds. Acute small brain lesions were found in a similar number of patients in each arm [apixaban 44/162 (27.2%); VKA 40/161 (24.8%); *P* = 0.64]. Cognitive function increased at the end of follow-up (median 1 MoCA unit; *P *= 0.005) without differences between study groups.

**Conclusions:**

Continuous apixaban is safe and effective in patients undergoing atrial fibrillation ablation at risk of stroke with respect to bleeding, stroke, and cognitive function. Further research is needed to reduce ablation-related acute brain lesions.

**Figure:**
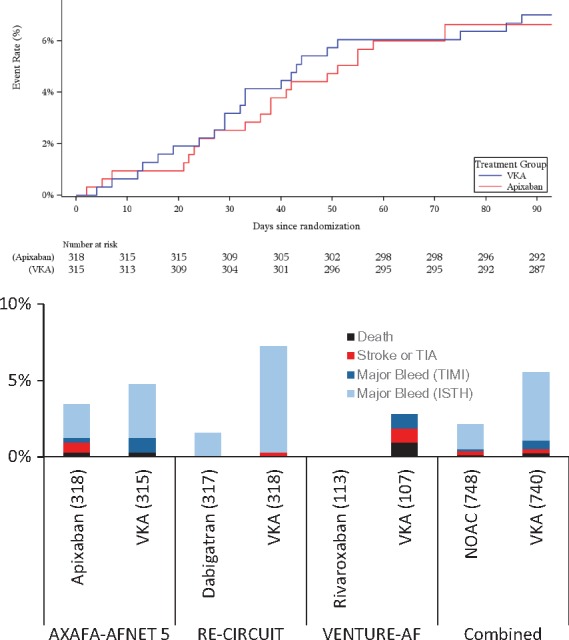

## Introduction

Catheter ablation is an effective^1–3^ and increasingly used component of rhythm control therapy to improve symptoms in patients with atrial fibrillation.^4–6^ Atrial fibrillation ablation is associated with a risk of stroke and major bleeding.^4–6^ Continuous oral anticoagulation using vitamin K antagonists (VKA) such as warfarin can reduce the risk of embolic events to <1% when combined with peri-procedural heparin.^7^ Therefore, continuous oral anticoagulation is recommended in patients undergoing atrial fibrillation ablation.^4^^,^^6^^,^^7^ One randomized trial comparing rivaroxaban to warfarin in 218 patients found similar bleeding rates with rivaroxaban compared to warfarin: 21/114 (18.4%) patients with bleeding on rivaroxaban, 18/104 (17.3%) patients with bleeding on VKA, one patient with stroke.^8^ Another trial randomizing 635 atrial fibrillation ablation patients to dabigatran or VKA found 59/318 (18.6%) patients with bleeding on dabigatran, 54/317 (17%) patients with bleeding on VKA, and one patient with transient ischaemic attack.^9^ Continuous apixaban has not been compared to VKA in atrial fibrillation ablation patients.

Atrial fibrillation ablation, unlike other ablation procedures, has been associated with declining cognitive function 90 days after the procedure, raising concerns about peri-procedural protection of the brain.^10^^,^^11^ Furthermore, acute brain lesions without corresponding neurological symptoms are detected in ca. 25% of patients undergoing atrial fibrillation ablation by high-resolution diffusion-weighted brain magnetic resonance imaging (MRI), a sequence that detects acute cytotoxic brain oedema.^12–15^ Cognitive function and acute brain lesions have not been evaluated in controlled clinical trials of patients undergoing atrial fibrillation ablation.

### Objectives

Therefore, we conducted a randomized trial comparing continuous apixaban to continuous VKA therapy in patients at risk of stroke undergoing atrial fibrillation ablation, including assessment of cognitive function in all patients and MRI-detected brain lesions in a sub-study.

### Trial design

*A*nticoagulation using the direct factor *Xa* inhibitor apixaban during *A*trial *F*ibrillation catheter *A*blation: comparison to VKA therapy (AXAFA – AFNET 5) was an investigator-initiated, prospective, parallel-group, randomized, open, blinded outcome assessment study comparing continuous apixaban therapy to VKA therapy. Details of the study design have been published.^16^ AXAFA – AFNET 5 was conducted in Europe and North America. The trial sponsor was AFNET, Münster, Germany (www.kompetenznetz-vorhofflimmern.de). AXAFA – AFNET 5 was designed by the steering committee in cooperation with AFNET and conducted in accordance with the declaration of Helsinki and the International Conference on Harmonization Good Clinical Practice Guidelines (ICH-GCP). The protocol was approved by ethics review boards at all institutions. The Clinical Research Institute (CRI, Munich, Germany) executed the study in cooperation with the steering committee and the sponsor. Data collection and entry were performed using the MARVIN^®^ eCRF system.^16–18^ An independent steering committee and an independent data and safety monitoring board guided the trial. All serious adverse events were adjudicated by an independent endpoint review committee blind to study group and international normalized ratio (INR) values. The Duke Clinical Research Institute served as the statistical core and performed the statistical analyses for the trial. The authors vouch for the accuracy and completeness of the data and for the fidelity of the trial to the protocol. This manuscript was written by the authors.

### Study population

AXAFA – AFNET 5 enrolled patients scheduled for a first atrial fibrillation ablation. Patients had at least one established stroke risk factor (age ≥ 75 years, heart failure, hypertension, diabetes, or prior stroke). The full inclusion and exclusion criteria have been published (see *Table 1*).^16^

**Table 1:** Inclusion and exclusion criteria of the AXAFA – AFNET 5 trial

Inclusion	Exclusion
Non-valvular atrial fibrillation (ECG-documented) with a clinical indication for catheter ablation	Any disease that limits life expectancy to <1 year
Clinical indication to undergo catheter ablation on continuous anticoagulant therapy	Participation in another clinical trial, either within the past 2 months or still ongoing
Presence of at least one of the CHADS_2_ stroke risk factors^a^	Previous participation in AXAFA
Age ≥ 18 years	Pregnant women or women of childbearing potential not on adequate birth control: only women with a highly effective method of contraception (oral contraception or intra-uterine device) or sterile women can be randomized
Provision of signed informed consent	Breastfeeding women
	Drug abuse or clinically manifest alcohol abuse
	Any stroke within 14 days before randomization
	Concomitant treatment with drugs that are strong dual inhibitors of cytochrome P450 3A4 (CYP3A4) and P-glycoprotein (P-gp) or strong dual inducers of CYP3A4 and P-gp
	Valvular AF (as defined by the focused update of the ESC guidelines on AF, i.e. severe mitral valve stenosis, mechanical heart valve). Furthermore, patients who underwent mitral valve repair are not eligible for AXAFA
	Any previous ablation or surgical therapy for AF
	Cardiac ablation therapy for any indication (catheter-based or surgical) within 3 months prior to randomization
	Clinical need for ‘triple therapy’ (combination therapy of clopidogrel, acetylsalicylic acid, and oral anticoagulation)
	Other contraindications for use of VKA or apixaban
	Documented atrial thrombi <3 months prior to randomization
	Severe chronic kidney disease with an estimated glomerular filtration rate (GFR) < 15 mL/min

Reproduced from the AXAFA – AFNET 5 design paper.^16^

a Stroke or TIA, age ≥ 75 years, hypertension, defined as chronic treatment for hypertension, estimated need for continuous antihypertensive therapy or resting blood pressure > 145/90 mmHg, diabetes mellitus, symptomatic heart failure (NYHA ≥ II).

### Treatment

At *baseline*, clinical parameters, stroke risk, heart rhythm, symptoms, quality-of-life (EQ5D, SF-12,^1^ and Karnofsky performance status^18^), and cognitive function [Montreal Cognitive Assessment Test (MoCA)]^19^ were assessed. Patients were randomized in a ratio of 1:1 to apixaban or VKA therapy. Randomization was stratified by study site and AF type (paroxysmal vs. persistent or long-standing persistent). The randomization scheme was generated via a computer programme using permuted block of a random size.

#### Apixaban

Patients randomized to apixaban received 5 mg b.i.d. throughout the study period. The apixaban dose was reduced to 2.5 mg b.i.d. if two or more of the following characteristics were present: age ≥80 years, body weight ≤60 kg, or serum creatinine level ≥1.5 mg/dL (133 μmol/L).^16^^,^^20^ Apixaban was continued during the ablation procedure without interruption, including on the morning of ablation. Continuous anticoagulation in this group was defined as having taken all but one apixaban dose per week based on pill count.

#### Vitamin K antagonist

Patients randomized to VKA were treated using the locally used VKA, e.g. warfarin, phenprocoumon, or acenocoumarol,^21^ prescribed and dispensed following local routine. Vitamin K antagonist therapy was monitored by INR measurements; a minimum of three INR measurements was mandatory prior to ablation. The last INR prior to ablation needed to be 1.8 or higher. The time in the therapeutic range was calculated by the Rosendaal method.^22^ Continuous anticoagulation in this group was defined by therapeutic INR (INR ≥ 2) in all INR measurements 30 days prior to catheter ablation.

All patients underwent follow-up visits at the time of the ablation procedure and 3 months after ablation. At the *ablation visit*, continuous anticoagulation for at least 30 days prior to ablation was assessed and an ECG performed. Transoesophageal echocardiography could be used following local practice. Interrupted anticoagulation required rescheduling of the ablation for 30 days unless (i) atrial thrombi were excluded by transoesophageal echocardiogram and (ii) effective anticoagulation was demonstrated prior to starting the ablation procedure by either taking at least two doses of apixaban (patients randomized to apixaban), or by an INR value ≥ 1.8 (patients randomized to VKA). A heparin bolus (100 IU/kg body weight) was required prior to or directly after transseptal puncture. The ablation procedure followed local practice and current guidelines.^4–6^ The protocol encouraged pulmonary vein isolation, the use of irrigated tip catheters, and flushing of all left atrial sheaths. Activated clotting time (ACT) was kept >300 s throughout the procedure. Activated clotting time measurements, details of the ablation technology used, delivered energy, procedure time, rhythm at beginning and end of procedure, and the need for cardioversion during the procedure were collected. An echocardiogram (transthoracic or intracardiac) was mandated directly after ablation to detect pericardial effusion.

At the 3 month visit, cognitive function and quality-of-life were reassessed, a 24 h Holter ECG was performed, and study medication was returned. A *final phone call* to assess serious adverse events was performed 30 days after discontinuation of study drug.

### Magnetic resonance imaging sub-study

Centres participating in the MRI sub-study (*n *= 25) offered brain MRI to all eligible study patients. A brain MRI was performed within 48 h after the ablation procedure. The MRI sequences were designed to detect all acute brain lesions, and to differentiate acute from chronic lesions. An imaging charta defined the MRI and adjudication workflow and brain MRI requirements (Supplementary material online, *Table S2*). The following MRI sequences were used: T2*-weighted imaging to screen for intracranial haemorrhage, diffusion-weighted imaging (DWI) and apparent diffusion coefficient (ADC) maps (post-processed) to assess acute brain infarction, and fluid-attenuated inversion recovery (FLAIR) to investigate the age of brain lesions.^14^^,^^15^ Diffusion-weighted imaging was conducted using a slice thickness of 2.5–3 mm (high-resolution DWI) to enhance the sensitivity of MRI for small lesions.^14^^,^^15^ Images failing the immediate quality check were repeated whenever feasible. All images were independently analysed by two experienced neuro-radiologists blinded to treatment allocation.

### Study outcomes

The *primary outcome* measured from randomization was the composite of all-cause death, stroke, or major bleeding among modified intention-to-treat (mITT) population, defined as all randomized patients who received study drug and underwent catheter ablation. Safety was assessed in all randomized patients receiving study drug (safety population). Sensitivity analyses were performed in all randomized patients (ITT). Another sensitivity analysis compared events during the peri-ablation period defined from ablation to 7 days after the procedure.^9^ Major bleeding was defined according to the Bleeding Academic Research Consortium (BARC ≥ 2).^23^ All bleeding events were centrally adjudicated according to the BARC, ISTH, and TIMI classifications.^23^^,^^24^

*Secondary outcomes* included time from randomization to ablation (ITT population), nights spent in hospital after ablation, ACT during ablation (summarized as median, 25th, 75th percentiles, and number of ACT measurements within the target range), all bleeding events, tamponade, need for transfusion, and changes in quality-of-life and cognitive function compared to baseline. In the MRI sub-study, the prevalence and number of MRI-detected acute brain lesions were compared between groups.

### Adverse events

All serious adverse events were collected, defined as adverse events that caused or prolonged hospitalization, caused disability or incapacity, were life-threatening, resulted in death or were important medical events. In addition, pregnancy, overdose, and cancer diagnosed after randomization were defined as serious adverse events. As AXAFA – AFNET 5 compared approved anticoagulants within their indications, non-serious adverse events were generally not reported, but those of special interest were defined and assessed. These comprised ablation-related complications including non-serious bleeding. The protocol encouraged brain imaging in patients who developed neurological abnormalities after the ablation procedure. All events from randomization to 3 months after index ablation procedure or to premature study termination were analysed.

### Statistical analysis

We estimated that a total of 650 patients (325 per group) were needed to detect a pre-specified margin of 7.5% (absolute difference) with 80% power using upper one-sided 95% confidence interval (i.e. two-sided 90% CI) with 3% attrition rate. The Farrington and Manning score test was used to compute sample size and power. The primary non-inferiority hypothesis was tested in the ablation population (mITT) using the method of Farrington and Manning score test with the pre-specified absolute margin of 0.075. In addition, a time-to-event analysis using Cox proportional hazards model with a relative margin of 1.44 was conducted. A multivariable Cox proportional hazards model controlling for the baseline risk factors of age, sex, weight, type of atrial fibrillation, and the CHADS_2_ factors was conducted. Changes in quality-of-life and cognitive function were assessed at 3 months compared to baseline using the EQ-5D and SF-12 questionnaires, MoCA, and Karnofsky scale. Changes in quality-of-life were compared by analysis of covariance (ANCOVA) models including the treatment arms as an indicator variable and the baseline quality-of-life variables as covariates. To accrue sufficient events for a formal non-inferiority analysis, AXAFA – AFNET 5 was exclusively conducted in patients at risk of stroke (*Table 1*) and counted bleeding events following the relatively broad BARC classification.^23^ An independent data and safety monitoring board monitored the study for safety. The Haybittle–Peto boundary was used as stopping rule guidance.

Descriptive statistics for continuous and categorical variables were summarized as means (SDs) and medians (25th, 75th percentiles), and numbers (percentages), respectively. Comparisons between continuous variables were performed using the Wilcoxon rank-sum test or two-sample *t*-test depending on normality; comparisons between nominal variables were performed using the Pearson’s χ^2^ test or Fisher’s exact test, depending on expected cell sizes. All analyses were two-sided and tested at the nominal 0.05 significance level. No adjustment was made for multiple testing. Statistical analyses were performed with SAS version 9.4 (SAS Institute Inc., Cary, NC, USA).

## Results

### Trial participants

**Figure 1:**
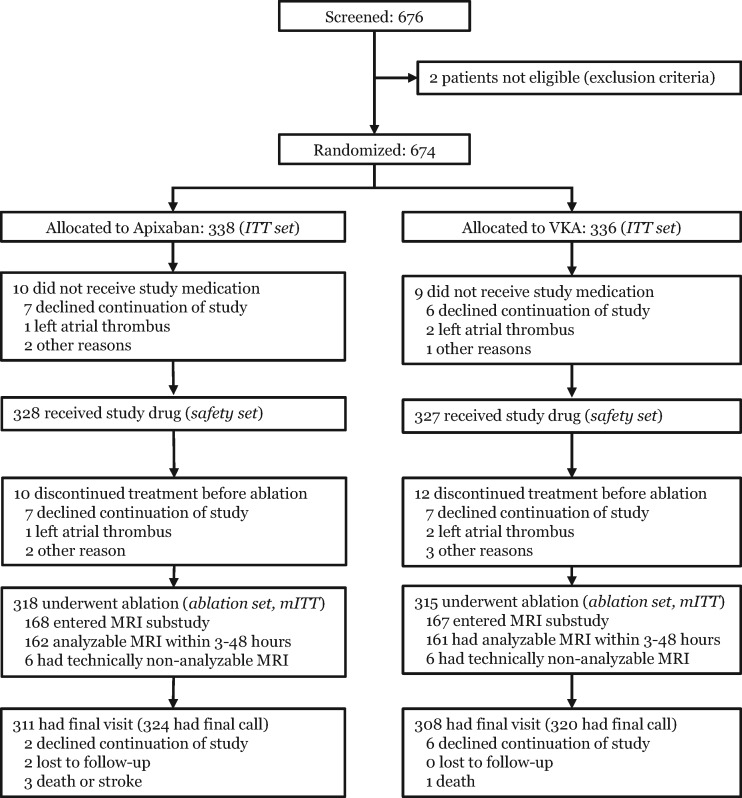
CONSORT diagram of the AXAFA – AFNET 5 study.

AXAFA – AFNET 5 randomized 674 patients across 49 sites in 9 countries from February 2015 to April 2017. Overall, 633 patients took study drug and underwent atrial fibrillation ablation (mITT, ablation set, *Figure 1*). Demographic and clinical characteristics were well balanced between groups (*Table 1*). Transoesophageal echocardiography was used in 549/633 (86.7%) patients. All or all but one apixaban doses per week were taken by 307/318 (97%) patients randomized to apixaban in the ablation set. The median time in therapeutic range in the 315 patients randomized to VKA in the ablation set was 84% (71, 97%). Time from randomization to ablation was not different between study groups (*Table 1*).

### Primary outcome

Primary outcome events (BARC 2–5 bleeding, stroke, or death) were observed in 22/318 (6.9%) patients randomized to apixaban, and in 23/315 (7.3%) patients randomized to VKA therapy in the ablation set. Four events were classified as TIMI major bleeding, and 24 events are ISTH major bleeding (*Table 3*). Two patients died: one patient randomized to VKA, female, age 70, hypertensive, last blood pressure 156/76, last INR 2.6, underwent pacemaker implantation 8 days after ablation and experienced a massive intracerebral haemorrhage. Another patient randomized to apixaban, male, age 69, with paroxysmal atrial fibrillation, hypertension, heart failure, diabetes, and chronic obstructive lung disease, was found dead in his bed 19 days after ablation without identifiable cause of death upon autopsy. Two patients randomized to apixaban had a stroke. Both had persistent AF and underwent transoesophageal echocardiogram. One patient, male, age 63, hypertensive, ACT 236–398 s, developed slurred speech with matching MRI lesion on the day of radiofrequency pulmonary vein isolation that fully resolved. Another patient, male, age 52, ACT 301–400 s, hypertensive, developed weakness of the right arm with paraesthesia of the right leg after cryoballoon pulmonary vein isolation that persisted beyond hospital discharge. Tamponade occurred in 2 (apixaban) and 5 (VKA) patients and was managed by pericardial drainage and administration of protamine and vitamin K. One patient with tamponade in each study group received blood transfusions. Anticoagulants were continued in five patients with tamponade, and paused for 4 days in one patient randomized to apixaban, and for 8 days in one patient randomized to VKA. All patients were discharged from hospital and attended the 3 months follow-up (*n* = 6) or an end of study visit (*n* = 1).

**Figure 2:**
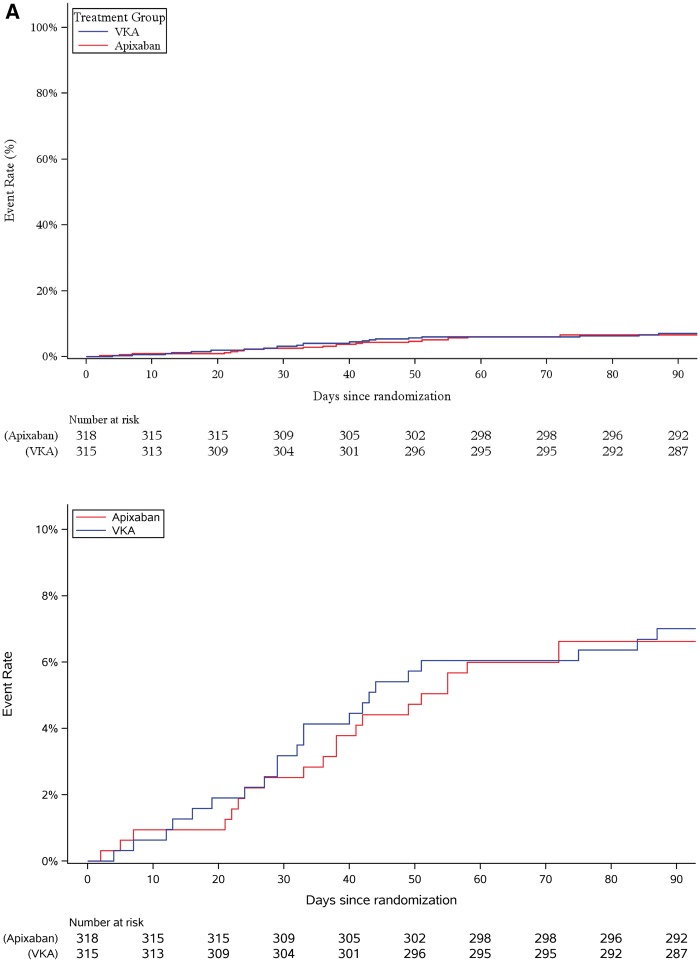

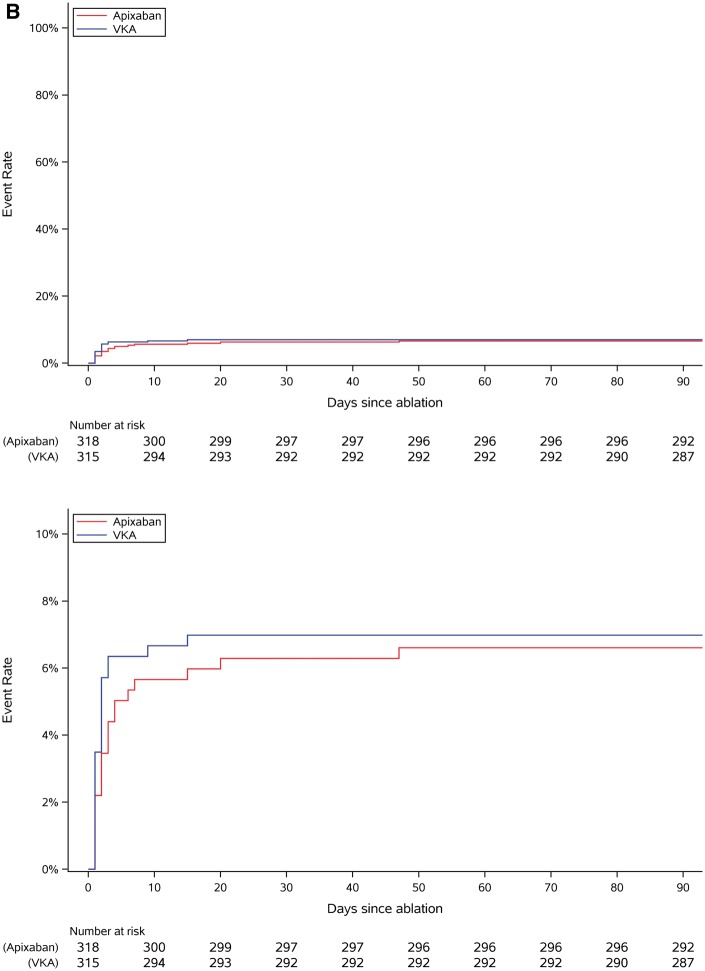
(*A*) Cumulative primary outcome events since randomization until 90 days after randomization at full scale (upper panel) and magnified (lower panel) in the ablation set. (*B*) Cumulative primary outcome events starting from ablation until 90 days after ablation at full scale (upper panel) and magnified (lower panel). VKA, vitamin K antagonist therapy.

**Figure 3:**
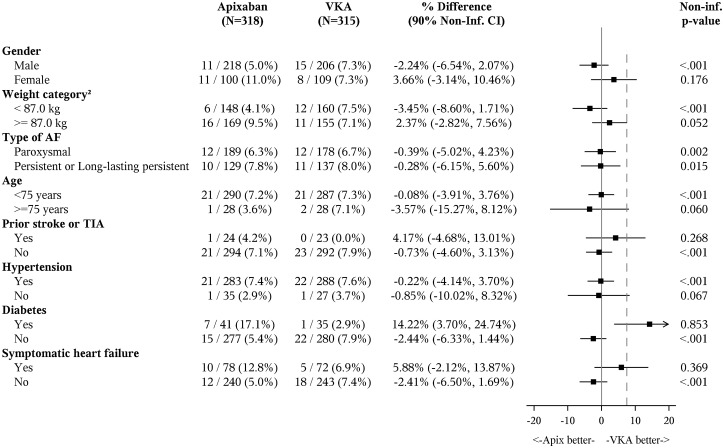
Forest plot of the differences (90% confidence intervals) in event rates in the main clinical subgroups. VKA, vitamin K antagonist therapy.

Apixaban was non-inferior to VKA based on the non-inferiority margin of 7.5% (a difference of −0.38%, 90% CI −4.0%, −3.3%, non-inferiority *P* = 0.0002). Apixaban was also non-inferior to VKA among all randomized patients as assessed by Cox proportional hazards model comparison between treatment groups using a relative non-inferiority margin of 1.44 (equivalent to 7.5% absolute; hazard ratio = 0.88, 90% CI 0.55, 1.41; *P* = 0.042, *Figure 2*). There was no statistical interaction between clinical stroke and bleeding risk factors and treatment groups (*Figure 3*).

### Secondary outcome parameters

There was no difference in time to ablation or nights spent in hospital after the ablation between groups (*Table 4*). As expected, the last INR prior to ablation and ACTs achieved during ablation were lower in the patients randomized to apixaban (*Table 4*). Quality-of-life as assessed by the physical component of SF-12 [+2.5 (−2.1, 8.1) units] and Karnofsky scale [+10 (0, 10)] improved during the study without differences between study groups (*Table 4*). At least mild cognitive dysfunction was found in 188/619 (30.4%) of the patients at baseline (pre-defined as MoCA < 26, *Table 2*). At the end of follow-up, MoCA increased by a median of +1.0 (−1.0, 2.0) unit without differences between study groups, and only 141/607 patients (7.2% fewer than at baseline) had mild cognitive impairment (*Table 4*).

**Table 2:** Clinical characteristics of the AXAFA – AFNET 5 ablation population

	All patients	Apixaban	VKA
	*n* = 633	*n* = 318 (*n* = 317 5 mg b.i.d., *n* = 1 2.5 mg b.i.d.)	*n* = 315 (*n* = 127 warfarin, *n* = 102 phenprocoumon, *n* = 86 acenocoumarol)
Age (years)			
Median age (q1, q3)	64 (58, 70)	64 (57, 70)	64 (58, 70)
Female sex	209 (33%)	100 (31%)	109 (35%)
Weight (kg)			
Median weight (q1, q3)	87.0 (77.0, 99.3)	88.0 (77.0, 100.0)	86.6 (76.0, 98.0)
Median body mass index (q1, q3)	28.3 (25.3, 31.6)	28.4 (25.5, 31.3)	28.2 (25.2, 31.9)
Concomitant conditions, stroke risk factors, and CHA_2_DS_2_-VASc score			
CHA_2_DS_2_-VASc score, mean (SD)	2.4 (1.2)	2.4 (1.2)	2.4 (1.2)
CHA_2_DS_2_-VASc score, median (q1, q3)	2 (2, 3)	2 (1, 3)	2 (2, 3)
Hypertension (*n*)	571 (90.2%)	283 (89.0%)	288 (91.4%)
Median systolic blood pressure (q1, q3)	138.0 (125.0, 150.0)	137.0 (125.0, 149.5)	140.0 (125.0, 152.0)
Median diastolic blood pressure (q1, q3)	82.0 (76.0, 90.0)	82.0 (75.0, 91.0)	82.0 (77.0, 90.0)
Symptomatic heart failure (NYHA II–IV)	150 (23.7%)	78 (24.5%)	72 (22.9%)
NYHA I	62 (9.8%)	30 (9.4%)	32 (10.2%)
NYHA II	126 (19.9%)	67 (21.1%)	59 (18.7%)
NYHA III	24 (3.8%)	11 (3.5%)	13 (4.1%)
NYHA IV	0	0	0
Diabetes mellitus	76 (12.0%)	41 (12.9%)	35 (11.1%)
Prior stroke or transient ischaemic attack	47 (7.4%)	24 (7.5%)	23 (7.3%)
Age ≥ 75 years	56 (8.8%)	28 (8.8%)	28 (8.9%)
Age 65–74 years	240 (37.9%)	122 (38.4%)	118 (37.5%)
Vascular disease, defined as coronary artery disease, peripheral artery disease, or carotid disease	83 (13.1%)	41 (12.9%)	42 (13.3%)
Valvular heart disease	73 (11.5%)	39 (12.3%)	34 (10.8%)
Mitral valve disease (moderate or more)	20	15	5
Aortic valve disease (moderate or more)	6	3	3
Confirmed coronary artery disease	77 (12.2%)	39 (12.3%)	38 (12.1%)
Chronic obstructive lung disease	39 (6.2%)	21 (6.6%)	18 (5.7%)
Clinical history of major bleeding	13 (2.1%)	10 (3.1%)	3 (1.0%)
Concomitant medical therapy			
*n* (%)	633	318	315
Amiodarone	102 (16.1%)	49 (15.4%)	53 (16.8%)
Dronedarone	13 (2.1%)	3 (0.9%)	10 (3.2%)
Flecainide	125 (19.7%)	59 (18.6%)	66 (21.0%)
Propafenone	16 (2.5%)	8 (2.5%)	8 (2.5%)
Sotalol > 160 mg/day	16 (2.5%)	7 (2.2%)	9 (2.9%)
ACE inhibitor or angiotensin receptor blocker	388 (61.3%)	192 (60.4%)	196 (62.2%)
Calcium channel antagonists	147 (23.2%)	72 (22.6%)	75 (23.8%)
Diuretics	221 (34.9%)	120 (37.7%)	101 (32.1%)
Antianginal medication	2 (0.3%)	0	2 (0.6%)
Antidiabetic medication	63 (10.0%)	32 (10.1%)	31 (9.8%)
Statins	231 (36.5%)	111 (34.9%)	120 (38.1%)
Platelet inhibitors or non-steroidal anti-inflammatory agents	30 (4.7%)	11 (3.5%)	19 (6.0%)
Beta blockers	451 (71.2%)	230 (72.3%)	221 (70.2%)
Digoxin or digitoxin	26 (4.1%)	17 (5.3%)	9 (2.9%)
Last INR before ablation (*n*)	531 (83.9)	217 (68.2%)	314 (99.7%)
Mean (SD)	1.9 (0.7)	1.2 (0.3), P < 0.001 vs. VKA	2.3 (0.5)
Median (q1, q3)	2.0 (1.1, 2.4)	1.1 (1.0, 1.2)	2.3 (2.0, 2.6)
Quality-of-life at baseline			
SF-12 physical component, *n* (%)	44.6 (37.7, 51.4), *n* = 597	43.5 (38.1, 51.3), *n* = 301	45.2 (37.6, 51.5), *n* = 296
SF-12 mental component *n* (%)	598 (94.5%)	301 (94.7%)	297 (94.3%)
SF-12 mental component *n* (%)	50.3 (42.8, 57.5), *n* = 598	51.2 (43.0, 57.9), *n* = 301	49.7 (42.6, 57.4), *n* = 297
Karnofsky scale	90 (80, 90)	80 (80, 90)	90 (80, 90)
Cognitive function [Montreal Cognitive Assessment (MoCA)] at baseline			
Median MoCA(q1, q3)	27.0 (25.0, 29.0), *n* = 618	27.0 (25.0, 29.0), *n* = 313	27.0 (25.0, 29.0), *n* = 305
At least mild cognitive impairment (MoCA < 26)	188 (30.4%)	93 (29.7%)	95 (31.1%)
Modified EHRA scale at baseline			
mEHRA I	40 (6.3%)	18 (5.7%)	22 (7.0%)
mEHRA IIa	164 (25.9%)	76 (23.9%)	88 (27.9%)
mEHRA IIb	205 (32.4%)	107 (33.6%)	98 (31.1%)
mEHRA III	208 (32.9%)	110 (34.6%)	98 (31.1%)
mEHRA IV	16 (2.5%)	7 (2.2%)	9 (2.9%)
Ablation information			
Atrial fibrillation pattern			
Paroxysmal atrial fibrillation	367 (58.0%)	189 (59.4%)	178 (56.5%)
Persistent or long-standing persistent atrial fibrillation	266 (42.0%)	129 (40.6%)	137 (43.5%)
Time from randomization to ablation (days)			
Mean (SD)	38.0 (27.3)	36.9 (27.6)	39.1 (27.0)
Median (q1, q3)	35.0 (20.0, 50.0)	34.0 (18.0, 48.0)	36.0 (21.0, 52.0)
Rhythm at start of ablation			
Number of patients	633	318	315
Sinus rhythm	434 (68.6%)	212 (66.6%)	222 (70.6%)
Atrial fibrillation	180 (28.4%)	98 (30.8%)	82 (26.0%)
Atrial flutter	12 (1.9%)	3 (0.9%)	9 (2.8%)
Pacing	7 (1.1%)	5 (1.6%)	2 (0.6%)
Other	0 (0%)	0 (0%)	0 (0%)
Type of ablation			
Pulmonary vein isolation, *n* (%)	571 (90.2%)	288 (90.6%)	283 (89.8%)
Pulmonary vein isolation plus other ablation, *n* (%)	59 (9.3%)	29 (9.1%)	30 (9.5%)
Other ablation without pulmonary vein isolation	3 (0.5%)	1 (0.3%)	2 (0.6%)
Transoesophageal echocardiography prior to ablation	549 (86.7%)	269 (84.6%)	280 (88.9%)
Total duration of ablation procedure (min), Median (q1, q3)	135 (110, 175)	136 (110, 175)	135 (105, 172)
Ablation energy source			
Radiofrequency	402 (63.5%)	207 (65.1%)	195 (61.9%)
Cryoablation	186 (29.3%)	92 (28.9%)	94 (29.8%)
Other	45 (7.1%)	19 (6.0%)	26 (8.3%)
Abnormal blood parameters			
Red blood cell count Abnormal	65/618 (10.5%)	32/311 (10.3%)	33/307 (10.7%)
Platelet count abnormal	35/625 (5.6%)	20/315 (6.3%)	15/310 (4.8%)
ALT abnormal	75/612 (12.3%)	39/307 (12.7%)	36/305 (11.8%)
Bilirubin abnormal	38/596 (6.4%)	14/297 (4.7%)	24/299 (8.0%)

Number of patients with valid information (*n* (%)) is only given when values were missing. BD, twice daily dosing; SD, standard deviation; q1, q3 are 25th and 75th percentiles, respectively; VKA, vitamin K antagonist.

**Table 3:** Primary outcomes in the AXAFA – AFNET 5 trial (ablation set), including details of the type of bleeding

	All patients	Apixaban	VKA
Patients with primary endpoint: composite of all-cause death, stroke or major bleeding	45/633 (7.1%)	22/318 (6.9%), non-inferiority *P*=0.0002	23/315 (7.3%)
Death	2 (0.3%)	1 (0.3%)	1 (0.3%)
Stroke or TIA	2 (0.3%)	2 (0.6%)	0
Major bleeding (BARC 2–5)	45 (7.1%)	20 (6.2%)	25 (7.9%)
Bleeding requiring medical attention (BARC 2)	24 (3.8%)	12 (3.7%)	12 (3.8%)
Bleeding with haemoglobin drop of 30 to <50 g/L or requiring transfusion (BARC 3a)	9 (1.4%)	5 (1.6%)	4 (1.3%)
Bleeding with haemoglobin drop ≥50 g/L, or requiring surgery or iv vasoactive agents, or cardiac tamponade (BARC 3b)	11 (1.7%)	3 (0.9%)	8 (2.5%)
Intracranial haemorrhage (BARC 3c)	1 (0.2%)	0	1 (0.3%, fatal)
TIMI major bleeding (Intracranial bleed, or bleeding resulting in a haemoglobin drop of ≥50 g/L, or bleeding resulting in death within 7 days)	4 (0.6%)	1 (0.3%)	3 (1%)
ISTH major bleeding	24 (3.8%)	10 (3.1%)	14 (4.4%)
Bleeding event by clinical type			
Tamponade	7 (1.1%)	2 (0.6%)	5 (1.6%)
Access site bleed	27 (4.3%)	12 (3.8%)	15 (4.8%)
Bleeding requiring transfusion of red blood cells	3 (0.5%)	2 (0.6%)	1 (0.3%)
Other major bleed	7 (1.1%)	5 (1.6%)	2 (0.6%)

Shown are number of patients per group. Some patients had more than one event. BARC4 events were not observed in the study.

b.i.d., twice daily dosing.

**Table 4:** Secondary outcomes in the AXAFA – AFNET 5 trial (ablation set)

	All patients	Apixaban	VKA
	*n* = 633	*n* = 318 (*n* = 317 5 mg BD, *n* = 1 2.5 mg BD)	*n* = 315 (*n* = 127 warfarin, *n* = 102 phenprocoumon, *n* = 86 acenocoumarol)
Time from randomization to ablation in days, median (q1, q3)	35.0 (20.0, 50.0)	34.0 (18.0, 48.0)	36.0 (21.0, 52.0)
Nights spent in hospital after index ablation, median (q1, q3)	3 (2, 5)	2 (1, 5)	3 (2, 7)
ACT during ablation in seconds, median (q1, q3)	325.0 (285.0, 370.0)	310.0 (273.0, 350.0)	348.5 (304.0, 396.0)
Number of subjects with all ACT values in range (*n* (%))	234/631 (37.1%)	73/316 (23.1%)	161/315 (51.1%)
Number of subjects with at least one ACT value < 250 (*n* (%))	214/631 (33.9%)	130/316 (41.1%)	84/315 (26.7%)
Number of subjects with at least one ACT value < 300 (*n* (%))	397/631 (62.9%)	243/316 (76.9%)	154/315 (48.9%)
Number of bleeding events (*n*)	118	54	64
Patients without recurrence of atrial fibrillation (*n* (%))	434/619 (70.1%)	217/311 (69.8%)	217/308 (70.5%)
Quality-of-life			
SF-12 physical component score at end of study, median (q1, q3), *n*	48.6 (42.0, 54.2), *n* = 564	48.4 (41.9, 54.2), *n* = 289	48.8 (42.2, 54.4), *n* = 275
Change in SF-12 physical component score at end of study compared to baseline, median (q1, q3), *n*	2.5 (−2.1, 8.1), *n* = 547, *P* < 0.001*	2.4 (−2.2, 7.9), *n* = 280	2.8 (−2.0, 8.3), *n* = 267
SF-12 mental component score at end of study, median (q1, q3), *n*	54.4 (46.0, 58.6), *n* = 565	54.2 (45.8, 58.3), *n* = 290	54.5 (46.6, 59.7), *n* = 267
Change in SF-12 mental component score at end of study compared to baseline, median (q1, q3), *n*	1.2 (−3.2, 8.0), *n* = 548, *P* < 0.001*	0.4 (−3.6, 8.0), *n* = 281	1.6 (−2.8, 8.3), *n* = 267
Karnofsky score at end of study, median (q1, q3), *n*	100 (90, 100), *n* = 619	100 (90, 100), *n* = 311	100 (90, 100), *n* = 308
Change in Karnofsky score at end of study compared to baseline (Δ Karnofsky), median (q1, q3),	10 (0, 10), *n* = 619	10 (0, 10), *n* = 311	10 (0, 10), *n* = 308
Cognitive function [Montreal Cognitive Assessment (MoCA)]			
Cognitive function at end of study (MoCA), median (q1, q3), *n*	28.0 (26.0, 29.0), *n* = 607	28.0 (26.0, 29.0), *n* = 305	28.0 (26.0, 29.0), *n* = 302
Abnormal MoCA at baseline (<26), *n* (%)	141 (23.2%)	75 (24.6%)	66 (21.9%)
Change in MoCA at end of study compared to baseline, median (q1, q3), *n*	1.0 (−1.0, 2.0), *n* = 597, *P* < 0.001*	0.0 (−1.0, 2.0), *n* = 301	1.0 (−1.0, 2.0), *n* = 296
Change in patients with abnormal MoCA at end of study compared to baseline, *n* (%)	141/607 (23.2%), −7.2%, *P* = 0.005*	75/305 (24.6%) −5.1%	66/302 (21.9%) −9.2%

Number of patients with valid information (*n* (%)) is only given when values were missing. *P-*values marked by asterisks (*) indicate differences between baseline and end of follow-up measurements. Twice daily (b.i.d.) dosing; q1 and q3 indicate 25th and 75th percentiles, respectively.

### Magnetic resonance imaging sub-study

**Figure 4:**
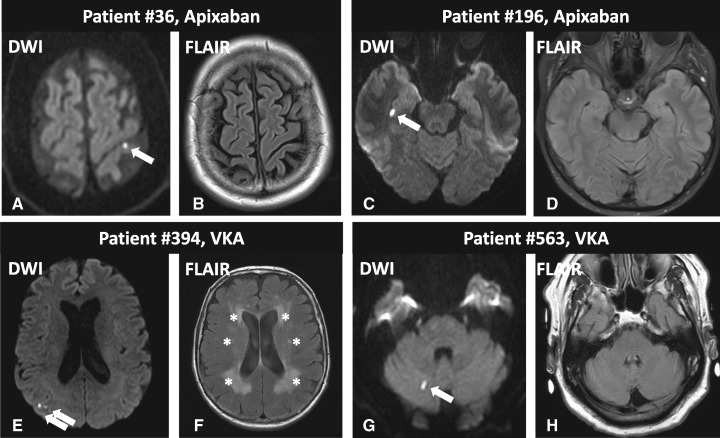
Examples of acute brain lesions detected in the brain magnetic resonance imaging sub-study. Acute brain lesions (arrows in *A*, *C*, *E*, *G*) are found by high-resolution diffusion-weighted brain magnetic resonance imaging without corresponding lesions in the fluid-attenuated inversion recovery images (arrows in *B*, *D*, *F*, *H*). Shown are representative lesions in two patients randomized to apixaban (*A*/*B*; *C*/*D*) and in two patients randomized to vitamin K antagonist (*E*/*F*; *G*/*H*). The fluid-attenuated inversion recovery images also detected chronic white matter lesions [asterisks (*) in F].

Acute brain MRI was performed in 335 patients across 25 centres. Clinical characteristics of the sub-study population were not different from the main study population, with the exception of a lower median weight in patients undergoing MRI [85.0 kg (74.5, 96.0)] compared to non-MRI patients [90.0 kg (80.0, 103.0)]. Clinical characteristics were well balanced between MRI sub-study treatment groups. There were 323 analysable MRIs. Acute brain MRI lesions (*Figure 4*) were found in 44/162 (27.2%) patients randomized to apixaban, and in 40/161 (24.8%) patients randomized to VKA (*P* = 0.635), with very similar distribution of lesions between random groups (*Table 5*). Cognitive function at the end of follow-up was not different in patients with or without acute brain lesions (MoCA 27.1 ± 2.7 in 239 patients without MRI lesions, 27.1 ± 2.8 in 84 patients with MRI lesions, *P* = 0.91).

**Table 5:** Acute brain lesions detected by high-resolution diffusion-weighted magnetic resonance imaging (MRI sub-study)

	All patients (*n* = 323)	Apixaban (*n* = 162)	VKA (*n* = 161)	*P*-value
No lesion	239 (74.0%)	118 (72.8%)	121 (75.2%)	0.635
Exactly one lesion	46 (14.2%)	27 (16.7%)	19 (11.8%)	0.211
Exactly two lesions	21 (6.5%)	7 (4.3%)	14 (8.7%)	0.111
More than two lesions	17 (5.3%)	10 (6.2%)	7 (4.3%)	0.463

*P*-values were determined by Pearson’ s χ^2^ test.

## Discussion

AXAFA – AFNET 5 demonstrated that continuous anticoagulation with apixaban is a safe and effective alternative to VKA in patients at risk of stroke undergoing atrial fibrillation ablation. AXAFA – AFNET 5 observed four TIMI major bleeding events in 633 patients (0.6%, *Table 3*) compared to one event in 248 patients in VENTURE-AF (0.4%).^8^ AXAFA – AFNET 5 observed 24 patients with ISTH major bleeding events (3.8%, *Table 3*) compared to 27 events in 635 patients in RE-CIRCUIT (4.3%).^9^ The numerical differences in ISTH major bleeding rates between AXAFA – AFNET 5 [apixaban 10 patients (3.1%); VKA 14 patients (4.4%); *Table 3*] and RE-CIRCUIT [dabigatran 5 patients (1.6%); VKA 22 patients (6.9%)]^9^ could be due to chance variations in outcomes, differences in risk profile between the AXAFA – AFNET 5 and RE-CIRCUIT study populations, and due to the high time in therapeutic range in the VKA group in AXAFA – AFNET 5 (median TTR 84%). AXAFA – AFNET 5 included only patients with stroke risk factors, resulting in a mean CHA_2_DS_2_VASc score of 2.4 and a population that was 4–5 years older than in the published controlled trials in atrial fibrillation ablation.^1–3^^,^^8^^,^^9^ Despite the higher stroke risk, we observed few strokes: AXAFA – AFNET 5 found 2 strokes in 633 patients (0.3%), compared to 1 stroke in 248 patients in VENTURE-AF (0.4%),^8^ and 1 TIA in 635 patients in RE-CIRCUIT (0.2%).^9^ Equally, mortality was low (0.3%) and similar to VENTURE-AF (0.4%),^8^ RE-CIRCUIT (0%),^9^ and the EORP AF ablation registry (0.2%).^25^

**Take home figure:**
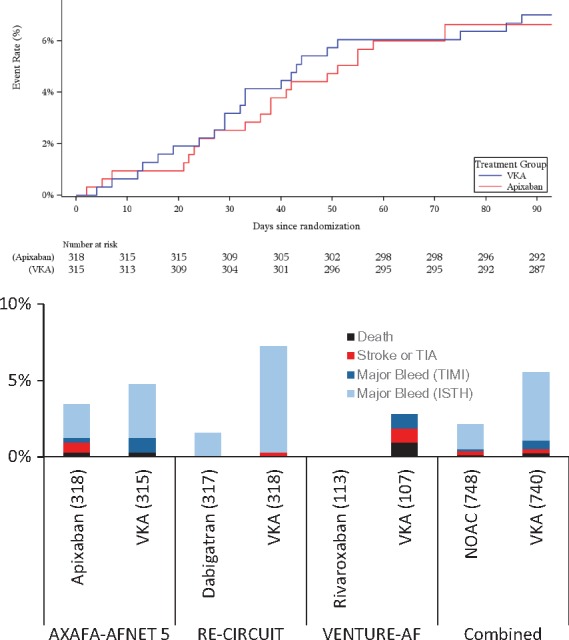
Cumulative outcome events in AXAFA – AFNET 5 in patients undergoing atrial fibrillation ablation at risk of stroke (top) and comparison to event rates in the two other controlled trials comparing continuous NOAC therapy with continuous vitamin K antagonist therapy (bottom). TIMI major bleeds were not separately reported in the main paper of RE-CIRCUIT. ISTH major bleeds were not separately reported in the main paper of VENTURE-AF.

AXAFA – AFNET 5 included 86 patients on acenocoumarol and 102 patients on phenprocoumon, 186 patients (29%) undergoing cryoablation,^3^ and 84 patients undergoing atrial fibrillation ablation without transoesophageal echocardiography without safety signals, providing some reassurance that these common patterns of clinical practice can be used on continuous apixaban or VKA therapy.^4–6^^,^^26^

The secondary outcomes observed in AXAFA – AFNET5 underpin the safety of continuous apixaban in atrial fibrillation ablation: time to ablation was not different between groups and quality-of-life and cognitive function improved equally in both study groups after ablation. High-resolution diffusion-weighted brain MRI detected acute brain lesions at the expected rate (∼25%)^12^^,^^14^^,^^15^ without differences between study groups. Continuous anticoagulation does not fully prevent acute brain lesions, which can be caused by debris dislodging from ablation wounds, air emboli, or small thrombi.^27^^,^^28^ Procedural improvements are desirable to reduce acute brain lesions during atrial fibrillation ablation.^29^ Further analyses of the AXAFA – AFNET 5 data set may shed more light on risk factors for acute brain lesions in patients undergoing AF ablation on continuous anticoagulation. One prior study found reduced cognitive function 90 days after atrial fibrillation ablation on interrupted warfarin therapy compared to baseline.^10^ Reassuringly, cognitive function improved at the end of AXAFA – AFNET 5 without differences between study groups.

### Limitations

AXAFA – AFNET 5 was an open study, but with blinded outcome assessment. The non-inferoirity margin was wide. The findings are consistent with prior studies with continuous dabigatran and rivaroxaban. While AXAFA – AFNET 5 was the first study comparing cognitive function after atrial fibrillation ablation in a controlled trial, the assessment was limited to global cognitive function. Differentiation between acute and chronic lesions was done by using an accepted combination of MRI sequences.^14^^,^^15^

## Conclusions

Continuous apixaban therapy is a safe and effective alternative to VKA in patients at risk of stroke undergoing atrial fibrillation ablation with respect to stroke, major bleeding, cognitive function, and MRI-detected acute brain lesions.

## Supplementary Material

Supplementary Data
