# Supplementary material for: Apixaban in patients at risk of stroke undergoing atrial fibrillation ablation
Source: Eur Heart J. 2018 Mar 20;39(32):2942–55. doi: 10.1093/eurheartj/ehy176 (PMC6110196; doi:10.1093/eurheartj/ehy176)

**Apixaban in patients at risk of stroke undergoing atrial fibrillation ablation**

**Online supplement: Committee members**

**Steering Committee:** Paulus Kirchhof (chair), Luigi Di Biase (co-chair), David Callans, Karl Georg Haeusler, Gerhard Hindricks, Hussein Al-Khalidi, Lluis Mont, Jens Cosedis Nielsen, Jonathan Piccini, Ulrich Schotten (non-voting, representing AFNET), Ma Hong (non-voting, representing Pfizer Inc, 235 42^nd^ Street, New York 10017, USA), Danny Liaw (non-voting, representing Bristol Myer Squibb, 100 Nassau Park Blvd, Princeton NJ 08543, USA)

**Data and Safety Monitoring Board:** Stefan Kääb^1^, Jan Tijssen^2^, Jose Zamorano^3^

**Endpoint Review Committee:** Julio Marti Almor^4^, Raymond Tukkie^5^, Roland Veltkamp^6^, Olav Wendelboe Nielsen^7^

**Core MRI lab experts:** Tobias Engelhorn^8^, Jochen B. Fiebach^9^

1. Ludwig-Maximilians-University, Munich, Germany
2. European Cardiovascular Research Institute, Amsterdam, Netherlands
3. University Hospital Ramon y Cajal, Madrid, Spain
4. Department of Cardiology and Coronary Unit, Barcelona, Spain
5. Spaarne Gasthuis, Haarlem, Netherlands
6. Faculty of Medicine, Imperial College, London, UK
7. Department of Clinical Medicine, University of Kopenhagen, Denmark
8. Department of Neuroradiology, University of Erlangen-Nuremberg, Erlangen, Germany
9. Center for Stroke Research Berlin, Charité – Universitätsmedizin Berlin, Germany

Affiliations of the steering committee members are given in the author list.

**Apixaban in patients at risk of stroke undergoing atrial fibrillation ablation**

**Online supplement: Sites and site PIs participating in AXAFA – AFNET 5**

Johan Vijgen, Jessa Ziekenhuis, Campus Virga Jesse, Belgium

Jim Hansen, Gentofte Hospital, Denmark

Massimo Grimaldi, Ospedale Generale Regionale F. Miulli, Italy

Arif Elvan, Isala Heart Centre, Zwolle, The Netherlands

Isabelle van Gelder, University Medical Center Groningen, The Netherlands

Gerhard Hindricks, Herzzentrum Leipzig, Germany

Tom De Potter, OLV Ziekenhuis Campus Aalst, Belgium

Jens Cosedis Nielsen, Århus Universitetshospital, Denmark

Daniel Scherr, Univ.-Klinik für Innere Medizin, Austria

Georg Nölker, Herz- und Diabeteszentrum NRW, Ruhr-Universität Bochum, Germany

Michael Block, Klinik Augustinum München, Germany

Lluis Mont, Hospital Clinic, Universitat de Barcelona, Spain

Steen Pehrson, Rigshospitalet Copenhagen, Denmark

Philippe Debruyne, Imeldaziekenhuis Bonheiden, Belgium

Sam Riahi, Aalborg Universitetshospital, Denmark

Jacob Pontoppidan, Odense Universitetshospital, Denmark

Philip Gentlesk, Sentara Cardiovascular Research Institute, United States

Andrea Sarkozy, Universitair Ziekenhuis Antwerpen, Belgium

Harry Crijns, University Hospital Maastricht, Netherlands

José Luís Merino, Hospital Universitario La Paz, Spain

Derick Todd, Liverpool Heart and Chest Hospital, Great Britain

Jippe Balt, St. Antonius Ziekenhuis Nieuwegein, Netherlands

Frank Provenier, AZ Maria Middelares, Belgium

Christopher Piorkowski, Herzzentrum Dresden Universitätsklinik, Germany

Andre Ng, University of Leicester, Glenfield General Hospital, Great Britain

Christopher Randall Ellis, Vanderbilt University Medical Center, United States

Tomas Szili Torok, Erasmus MC Rotterdam, Netherlands

Markus Stühlinger , Medizinische Universität Innsbruck, Austria

Thomas Arentz, Universitäts-Herzzentrum Freiburg Bad Krozingen, Germany

Luigi Di Biase, Montefiore Medical Center - Univ. Hospital for A. Einstein Coll. of Medicine, United States

Anja Dorszewski, Herzzentrum Duisburg, Evangelisches Klinikum Niederrhein gGmbH, Germany

Thomas Rostock, Universitätsmedizin der Johannes Gutenberg-Universität Mainz, Germany

Julián Pérez Villacastín, Hospital Clínico Universitario San Carlos, Spain

Joseph De Bono, Queen Elisabeth Hospital Birmingham, Great Britain

Andrea Natale, Texas Cardiac Arrhythmia Research Foundation , United States

Richard Schilling, St. Bartholomew's Hospital, Great Britain

Emanuele Bertaglia, Azienda Ospedaliera di Padova, Italy

Andreas Götte, St. Vincenz-Krankenhaus Paderborn, Germany

Helmut Pürerfellner, Krankenhaus der Elisabethinen Linz GmbH, Austria

Jan Schrickel, Universitätsklinikum Bonn, Germany

Giuseppe Stabile, Clinica Mediterranea di Napoli, Italy

Clemens Steinwender, Kepler Universitätsklinikum, Med Campus III, Austria

Daniel Steven, Herzzentrum der Universität zu Köln, Germany

Sakis Themistoclakis, Ospedale Dell'Angelo, Italy

Stephan Willems, Universitäres Herzzentrum Hamburg, Germany

Leif-Hendrik Boldt, Charité Berlin, Campus Virchow-Klinikum, Germany

David Callans, Hospital of the University of Pennsylvania, United States

Peter Loh, UMC Utrecht, Hart Long Centrum, Netherlands

Boris Rudic, Universitätsmedizin Mannheim, Germany

**Apixaban in patients at risk of stroke undergoing atrial fibrillation ablation**

**Online supplement: Sites and lead radiologists participating in the MRI sub-study within AXAFA – AFNET 5**

Hannes Deutschmann, Medizinische Universität Graz, Graz, Austria

Andreas Rietzler, Medizinische Universität Innsbruck, Innsbruck, Austria

Manfred Gschwendtner, Krankenhaus der Elisabethinen Linz, Linz, Austria

Piet Vanhoenacker, OLV Ziekenhuis Campus Aalst, Aalst, Belgium

Annick Demeyere, Imeldaziekenhuis Bonheiden, Bonheiden, Belgium

Stefan Palmers, AZ Maria Middelares, Gent, Belgium

Geert Souverijns, Jessa Ziekenhuis, Campus Virga Jesse, Hasselt, Belgium

Won Yong Kim, Århus Universitetshospital, Aarhus, Denmark

Dimitar Ivanov Radev, Hospital Gentofte, Hellerup, Denmark

Gregor Pache, Universitäts-Herzzentrum Freiburg Bad Krozingen , Bad Krozingen, Germany

Hermann Esdorn, Herz- und Diabeteszentrum NRW, Ruhr-Universität Bochum, Bad Oeynhausen, Germany

Marietta Garmer, Grönemeyer Institut für MikroTherapie, Bochum, Germany

Jens Fiehler, Universitätsklinikum Hamburg-Eppendorf, Hamburg, Germany

Matthias Gutberlet, Herzzentrum Leipzig, Leipzig, Germany

John Morlese, University of Leicester, Glenfield General Hospital, Leicester, Great Britain

James Moon, St. Bartholomew's Hospital , London, Great Britain

Maria Gentile, Ospedale Generale Regionale F. Miulli, Acquaviva delle Fonti, Italy

Hendrik van Es, St. Antonius Ziekenhuis Nieuwegein, EM Nieuwegein, Netherlands

Linda Jacobi-Postma, University Hospital Maastricht, Maastricht, Netherlands

Tim Leiner, UMC Utrecht, Utrecht, Netherlands

Geert Jan Waldman, Isala Diagram B.V. SMO Zwolle, Zwolle, Netherlands

Núria Bargalló, Hospital Clinic, Universitat de Barcelona , Barcelona, Spain

Deb Cardinal, Texas Cardiac Arrhythmia Research Foundation , Austin, United States

Jeff Creasy, Vanderbilt University Medical Center, Nashville, United States

Judah Burns, Montefiore Medical Center - Univ. Hospital for A. Einstein Coll. of Medicine, New York, United States

**Apixaban in patients at risk of stroke undergoing atrial fibrillation ablation**

**Supplementary Table 1:** Imaging criteria for acute brain magnetic resonance imaging.

| **Sequence** | **Sequence type** | **Repetition time** | **Echo time** | **Averages**  **b-values** | **Field-of-view** | **Acquisition**  **matrix** | **Slice thickness** | **Gap** | **Slices coverage** |
| --- | --- | --- | --- | --- | --- | --- | --- | --- | --- |
| Standard diffusion-weighted imaging | Spin echo -Echo planar imaging | Local standard | Local standard | b = 0  b = 1000 | Local standard | Local standard | 5 - 6 mm | 0 | 20 - 25/  whole brain |
| High resolution diffusion-weighted imaging or diffusion tensor imaging | Spin echo -Echo planar imaging | > 8000 | 1.5 Tesla:  < 120  3.0 Tesla:  < 100 | b = 0  1-2 Averages  b = 1000  6 Averages | 220 - 300 mm | 1.5 Tesla:  ≥ 128x128  3.0 Tesla:  ≥ 192x128 | 2.5 – 3 mm | 0 | 40-50/  whole brain |
| Fluid attenuated inversion recovery | Inversion recovery | > 8000  Inversion time: scanner depended |  | 1 | 220 - 300 mm | 1.5 Tesla:  ≥ 192x128  3.0 Tesla:  ≥ 256x192 | 5 – 6 mm | max 10% | 20-25/  whole brain |
| T2*, Susceptibility weighted imaging or the like | Gradient echo | 620 | 20 | 1 | 220 - 300 mm | 1.5 Tesla:  ≥ 192x128  3.0 Tesla:  ≥ 256x192 | 5 – 6 mm | max 10% | 20-25/  whole brain |

**Apixaban in patients at risk of stroke undergoing atrial fibrillation ablation**

**Supplementary Information: Change management of anticoagulants at start and discontinuation of study drug in the AXAFA – AFNET 5 trial.** This information was included as an appendix in the protocol of the AXAFA – AFNET 5 trial.


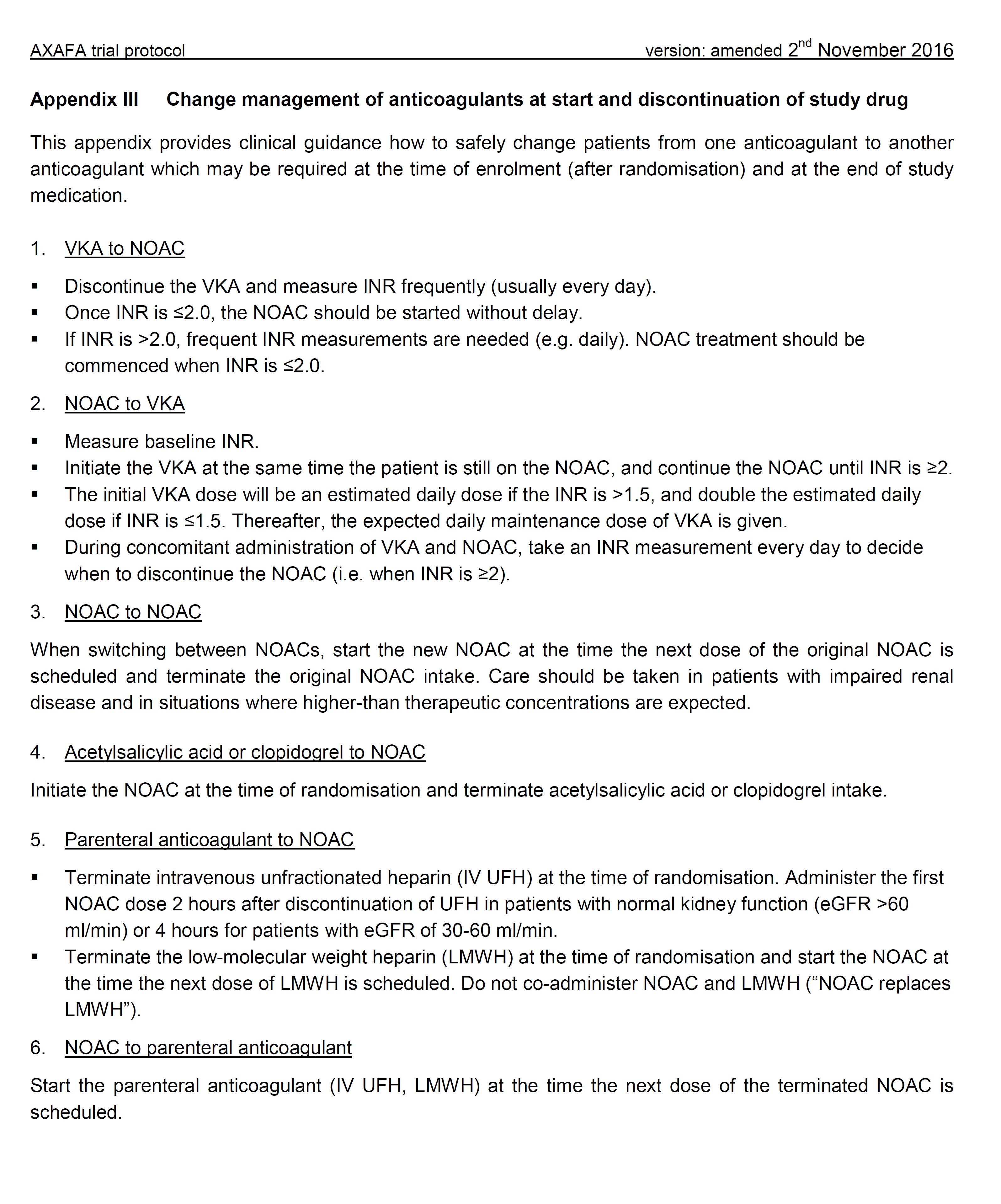

Supplement: Supplementary Data [file ehy176_supplementary_material.docx]
